# Titration of extra-PEEP against intrinsic-PEEP in severe asthma by electrical impedance tomography: A case report and literature review

**DOI:** 10.1097/MD.0000000000020891

**Published:** 2020-06-26

**Authors:** Huaiwu He, Siyi Yuan, Chi Yi, Yun Long, Rui Zhang, Zhanqi Zhao

**Affiliations:** a Department of Critical Care Medicine, Peking Union Medical College Hospital, Chinese Academy of Medical Sciences, Dongcheng District, Beijing; b Department of Biomedical Engineering, Fourth Military Medical University, Xi’an, China; c Institute of Technical Medicine, Furtwangen University, Villingen-Schwenningen, Germany.

**Keywords:** electrical impedance tomography, extra-positive end-expiratory pressure, occult pendelluft phenomenon, regional ventilation delay, severe asthma

## Abstract

**Rationale:**

The use of extra-positive end-expiratory pressure (PEEP) at a level of 80% intrinsic-PEEP (iPEEP) to improve ventilation in severe asthma patients with control ventilation remains controversial. Electrical impedance tomography (EIT) may provide regional information for determining the optimal extra-PEEP to overcome gas trapping and distribution. Moreover, the experience of using EIT to determine extra-PEEP in severe asthma patients with controlled ventilation is limited.

**Patients concerns:**

A severe asthma patient had 12-cmH_2_O iPEEP using the end-expiratory airway occlusion method at Zero positive end-expiratory pressures (ZEEP). How to titrate the extra-PEEP to against iPEEP at bedside?

**Diagnoses and Interventions:**

An incremental PEEP titration was performed in the severe asthma patient with mechanical ventilation. An occult pendelluft phenomenon of the ventral and dorsal regions was found during the early and late expiration periods when the extra-PEEP was set to <6 cmH_2_O. If the extra-PEEP was elevated from 4 to 6 cmH_2_O, a decrease in the end-expiratory lung impedance (EELI) and a disappearance of the pendelluft phenomenon were observed during the PEEP titration. Moreover, there was broad disagreement as to the “best” extra-PEEP settings according to the various EIT parameters. The regional ventilation delay had the lowest extra-PEEP value (10 cmH_2_O), whereas the value was 12 cmH_2_O for the lung collapse/overdistension index and 14 cmH_2_O for global inhomogeneity.

**Outcomes:**

The extra-PEEP was set at 6 cmH_2_O, and the severe whistling sound was improved. The patient's condition further became better under the integrated therapy.

**Lessons:**

A broad literature review shows that this was the 3rd case of using EIT to titrate an extra-PEEP to against PEEPi. Importantly, the visualization of occult pendelluft and possible air release during incremental PEEP titration was documented for the first time during incremental PEEP titration in patients with severe asthma. Examining the presence of the occult pendelluft phenomenon and changes in the EELI by EIT might be an alternative means for determining an individual's extra-PEEP.

## 1 Introduction

The use of extra-positive end-expiratory pressure (PEEP) vs intrinsic-PEEP (iPEEP) in severe asthma patients under control ventilation remains controversial. An extra-PEEP level that is too low can lead to small airway collapse and air trapping, whereas extra-PEEP values that are too high could cause further lung hyperinflation and circulation compromise.^[1]^ An extra-PEEP value of 80% that of the iPEEP is suggested to open the small airway and reduce hyperinflation.^[2]^ However, one size does not fill all. Individualized responses to extra-PEEP have been found in patients with airway obstruction during controlled ventilation.^[3]^ The extra-PEEP settings for severe asthma during controlled ventilation remains challenging in clinical practice. Electrical impedance tomography (EIT) is a noninvasive, radiation-free imaging tool that has generated great interest for the mechanical ventilation of critically ill patients, and could provide dynamic information on the heterogeneity of ventilation (inflation/deflation) and lung volumes under different clinical conditions.^[4–6]^

Hence, EIT monitoring might be useful for determining individual extra-PEEP settings in severe asthma patients under control ventilation. Here, we report a case study and literature review of PEEP titration guided by EIT in severe asthma patients.

## 2 Case presentation

A 84-year-old woman with severe asthma was intubated and mechanically ventilated due to respiratory failure. She was sedated and paralyzed and administered volume-controlled ventilation with a constant inspiratory flow of 30 L/min, which resulted in an inspiratory: expiratory period ratio of 1:5 (tidal volume 360 mL and respiratory rate 13 breaths/min). An intrinsic positive end-expiratory pressure (iPEEP) of 12 cmH_2_O was confirmed using the end-expiratory airway occlusion method when the extra-PEEP was set to zero. Extra-PEEP titration was performed from 0 to 14 cmH_2_O with steps of 2 cmH_2_O.

The ventilation distribution was closely monitored with EIT during the incremental PEEP titration. The related EIT parameters (collapsed and overdistention percentage [CL%/OD%], global inhomogeneity (GI), regional ventilation delay (RVD), standard deviation of RVD (SD-RVD), and center of ventilation (CoV) index) from the incremental PEEP titration were analyzed offline according to a previously described EIT-based algorithm.^[4]^ The EIT-based “best” extra-PEEP was defined as the lowest value of EIT-derived parameters (CL/OD index, GI, RVD, and CoV) with the lowest pressure level.

**Figure 1:**
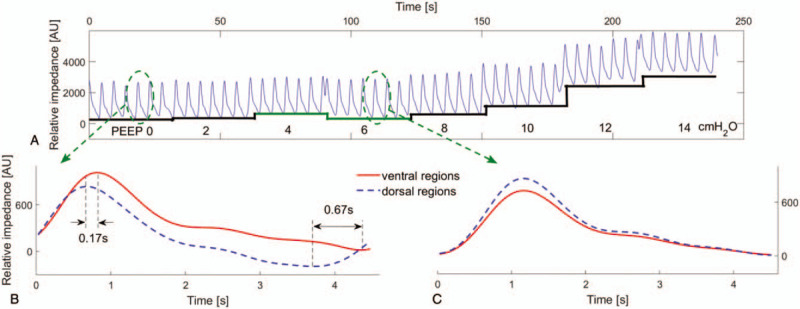
(A) Global impedance curves during the incremental positive end-expiratory pressure (PEEP) titration from 0 to 14 cmH_2_O. The end-expiratiory lung impedance continously increase from 0 to 14 cmH_2_O with an exception from 4 to 6 cmH_2_O (green solid line). (B) The occult pendelluft phenomenon was oberseved at PEEP 0 cmH_2_O. The early deflation in the dorsal regions was accompanied by concomitant inflation of ventral regions at the early expiration (the time difference of deflation start between dorsal and ventral regions was 0.17 second). The early inflation in the dorsal regions was accompanied by concomitant deflation of ventral regions at the late expiration (the time difference of deflation start between dorsal and ventral regions was 0.67 second). (C) Simultaneous deflation of the dorsal and ventral regions was observed at PEEP 6 cmH_2_O. Red solid lines, relative impedance changes from ventral regions; blue dashed lines, relative impedance changes from dorsal regions.

**Figure 2:**
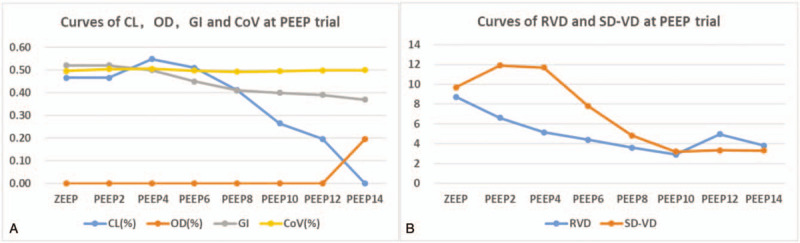
(A) Changes curves of collapsed (CL), overdistention (OD), global inhomogeneity (GI), and center of ventilation index (CoV) during the incremental positive end-expiratory pressure (PEEP) trial. (B) Change curves of regional ventilation delay (RVD) and standard deviation of regional ventilation delay (SD-VD) during the incremental PEEP trial.

A drop of global end-expiratory lung impedance (EELI) at an extra-PEEP of 6 cmH_2_O was found when compared to 4 cmH_2_O (Fig. 1A, green solid line). Additionally, desynchrony of deflation in the ventral and dorsal regions was observed when the PEEP levels were <6 cmH_2_O in this case. Specifically, the occult pendelluft phenomenon was found at the early and late expiration periods. The early inflation in the dorsal regions was accompanied by concomitant deflation in the ventral regions at late expiration (Fig. 1B). The early deflation in the dorsal regions was accompanied by concomitant inflation in the ventral regions at early expiration (Fig. 2B). When the extra PEEP was adjusted to levels ≥6 cmH_2_O, simultaneous deflation of the ventral and dorsal regions was observed (Fig. 1C). Additionally, the occult pendelluft phenomenon also disappeared. Moreover, a severe whistling sound was captured via bedside auscultation at PEEP values <6 cmH_2_O when compared to PEEP values ≥6 cmH_2_O. Finally, the extra-PEEP was set at 6 cmH_2_O in this patient.

**Figure 3:**
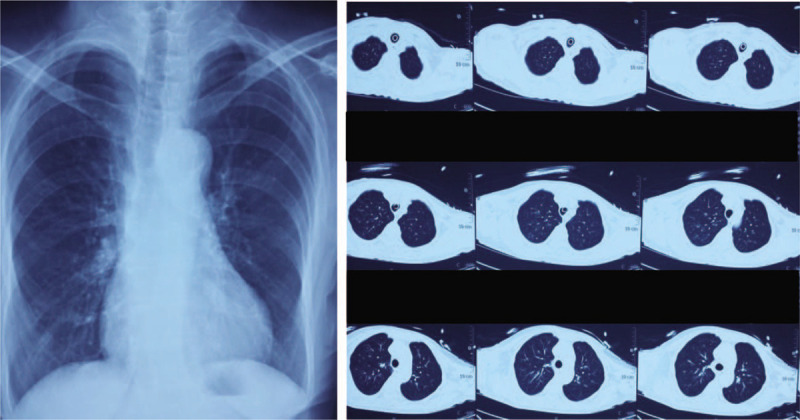
Chest X-ray and computed tomography scan.

Measurements of lung mechanics and EIT-related parameters at different PEEP levels are shown in Table 1. There was broad disagreement in the various “best” extra-PEEP values according to the different EIT parameters. The RVD and SD-RVD had the lowest values at an extra-PEEP setting of 10 cmH_2_O, whereas the lowest values for the lung CL/OD index and GI index 12 and 14 cmH_2_O, respectively. There was no a significant change in the CoV during the PEEP titration. These GI, CL, OD, CoV, RVD, and SD-RVD curves are shown in Figure 2. The patients’ chest X-ay and computed tomography scan are shown in Figure 3.

**Table 1:**
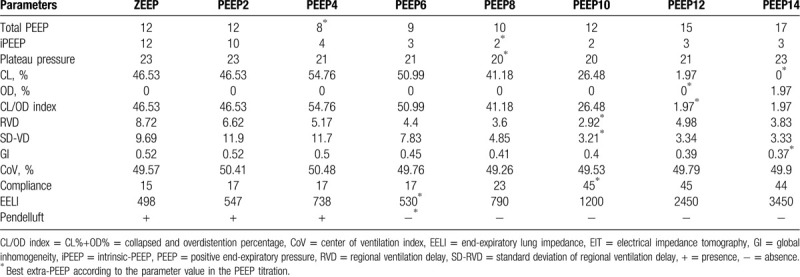
Changes of lung mechanics and EIT-related parameters during incremental PEEP titration.

### 2.1 Literature review

A systematic literature search was conducted in PubMed on articles in English using the following search terms: “EIT” and “severe asthma” or “asthma” or “COPD” or “PEEPi” or “mechanical ventilation” or “extra-PEEP” or “flow limitation.” After selections, only 2 articles reported that using EIT to titrate extra-PEEP in the mechanical ventilated patients with PEEPi.^[7,8]^ To best our knowledge, this was the 3rd case of using EIT to titrate an extra-PEEP to against PEEPi.

## 3 Discussion

An unified extra-PEEP (80% iPEEP) is suggested to offset the critical airway closing pressure during mechanical ventilation for severe asthma. Recently, 2 cases have been reported using EIT technology to adjust individual extra-PEEP settings in severe asthma patients. In 2013, Mauri et al revealed that the extra-PEEP could be set to 120% of the iPEEP according to the highest regional iPEEP assessed using EIT.^[7]^ Moreover, Kostakou et al reported that an 80% iPEEP resulted in the best delay ventilation (RVD) as determined by EIT.^[8]^

In the present patient, occult pendelluft and a decrease in the EELI (indicating the release of air) was observed with EIT during the incremental PEEP titration. Yoshida T et al^[9]^ reported that spontaneous breathing could induce the occult pendelluft phenomenon in anesthetized pigs, which could be observed with EIT. The pendelluft phenomenon might be caused by regional deflation desynchrony and pressure imbalance. Interestingly, the release of air (decreased EELI) was accompanied by the absence of the expiratory pendelluft when the extra-PEEP was set to 6 cmH_2_O. Hence, an extra-PEEP of 6 cmH_2_O (equal to 50% iPEEP) improved the flow limitation and reduced hyperinflation. To the best of our knowledge, this is the first report demonstrating the occult pendelluft phenomenon in a sedated, paralyzed severe asthma patient under controlled ventilation. Marui et al^[7]^ reported that air trapping could be detected by EIT during a decremental external-PEEP titration in a patient with chronic obstructive pulmonary disease. Caramez et al found that proper external-PEEP application may relieve overinflation in select patients with airway obstruction during controlled mechanical ventilation.^[3]^ However, the visualization of possible air release during incremental PEEP titration was documented for the first time in the present report.

Moreover, there was significant variation in the “best” extra-PEEP setting according to the different EIT methods/parameters during the PEEP titration. Since expiration flow limitation is the main pathophysiologic mechanism of severe asthma, the CL/OD and RVD might be potential methods for assessing the regional ventilated condition.^[10]^ An inflection point was observed on the CL/OD and RVD curves but not on the GI and CoV curves, indicating that using the CL/OD and RVD could reflect the risk/benefits of extra-PEEP vs iPEEP. Hence, using PEEP titration to determine an individual's extra-PEEP setting compared to iPEEP might be necessary in clinical practice. According to the best regional tidal compliance at different PEEP levels, the percentage of alveolar collapse and overdistension was defined using the CL/OD method. In the present study, the percentage of alveolar collapse continuously decreased, with a very limited increase in alveolar overdistension during the incremental PEEP titration (Fig. 2A). Actually, the decrease in alveolar collapse was due to reopening of the small airways, not reopening of the collapsed alveolar when the PEEP setting was increased in this patient. Further studies are required to validate which EIT parameters/methods should be used to titrate to the best extra-PEEP to improve patent outcomes. Studies had reported different responses to various EIT-derived parameters during decremental PEEP titration in ARDS patients.^[10,11]^ Here, we emphasize that attention should be paid to the occult pendelluft phenomenon and decreases in the EELI during incremental PEEP titration in severe asthma patients with controlled ventilation. Moreover, the CL/OD and RVD might be helpful for determining the best extra-PEEP setting when the occult pendelluft phenomenon is absent and the EELI decreases during incremental PEEP titration.

## 4 Conclusion

Examining the presence of the occult pendelluft phenomenon and changes in the EELI by EIT might be an alternative means for determining an individual's extra-PEEP value during incremental PEEP titration in patients with severe asthma. However, additional studies are required to validate which EIT parameters/methods could lead to better outcomes.

## Author contributions

**Conceptualization:** Huaiwu He, Chi Yi, Yun Long, Rui Zhang, Zhanqi Zhao.

**Data curation:** Chi Yi, Huaiwu He.

**Formal analysis:** Siyi Yuan, Huaiwu He, Rui Zhang.

**Investigation:** Huaiwu He, Chi Yi.

**Methodology:** Siyi Yuan, Chi Yi, Rui Zhang.

**Software:** Huaiwu He, Zhanqi Zhao.

**Supervision:** Yun Long, Huaiwu He.

**Writing – original draft:** Huaiwu He, Siyi Yuan, Chi Yi, Yun Long, Rui Zhang, Zhanqi Zhao.

**Writing – review & editing:** Huaiwu He, Siyi Yuan, Chi Yi, Rui Zhang, Zhanqi Zhao.

## Correction

This article was originally published with an incorrect reference. Reference 8 has been corrected from Blankman P, Hasan D, Erik G, et al. Detection of ’best’ positive endexpiratory pressure derived from electrical impedance tomography parameters during a decremental positive end-expiratory pressure trial. Crit Care 2014;18:R95. to Kostakou E, Barrett N, Camporota L. Electrical impedance tomography to determine optimal positive end-expiratory pressure in severe chronic obstructive pulmonary disease.Crit Care. 2016. 22;20(1):295.
